# 

**Published:** 1902-04-26

**Authors:** 


### NOTICE TO CORRESPONDENTS.

All MSS., Letters, Books for Review, and other matters intended for the Editor should be addressed The Editor, "The Hospital," The Hospital Building, 28 & 29 Southampton Street, Strand, W.O.
The Editor cannot undertake to return rejected MS., even when accompanied by stamped directed envelope.

**Notice to Advertisers and Subscribers.**

All Advertisements, Orders for Copies of The Hospital, and Business Communications should be addressed to **THE MANAGER, (Not to the Editor)**, The Hospital, 28 & 29 Southampton Street, Strand, London, W.O.

The Cost of Subscription, post free, is as follows.—Per week, 2½d.; per quarter, 2s. 8d.; per half-year, 5s. 3d.; per annum, 10s. 6d. Foreign:—Per annum, 15s. 2d., payable, in all cases, in advance.

**NOTICE.—Vol. XXXI. of "The Hospital" (October 1901, to March 1902), in handsome cloth binding will shortly be on sale at the office of this Journal, price, 7s. 6d. Cases for binding the Numbers contained in this Volume 1s. 6d. Index to Vol. XXXI., 2½d., post free.**
**The Monthly Part for May will be ready on May 1. Price 6d., by post 8d.**

### SCRAPS AND GLEANINGS.

THE new buildings at Western Bank of the Sheffield Children's Hospital are progressing rapidly.

IT is anticipated that the cost of the new building of the Manchester Southern Hospital will be £35,000.

WELFORD AND SONS, LIMITED, Elgin Avenue, London, W., have been honoured with a royal warrant appointing them dairymen to His Royal Highness the Prince of Wales.

A MEETING of ladies was held recently at Bute House, under the presidency of Mrs. Bischoffsheim, to promote the success of the appeal now being made for £50,000 for the enlargement of the North-Eastern Hospital for Children.

AT the meeting of ratepayers recently held to protest against the scheme of the Bristol guardians for the erection of a new workhouse infirmary at a cost of upwards of £200,000, the voting was as follows:—For the resolution of protest, 52; against, 90. The resolution was therefore lost.

THE financial statement presented at the annual meeting of the Bolton Infirmary recently showed that during the past year the income was £6,367 and the expenditure £6,771, leaving a deficit of £404. There was a falling off of £180 in the Hospital Saturday collections.

THE annual statement of accounts of Peterborough Infirmary shows that the total income for the year was £2,260 odd, as against £2,097 in 1900; but the expenditure had also increased and the accounts showed a deficit of £89 as against £65 the previous year.

DURING 1901 there were 356 in-patients treated at the Manchester Southern Hospital and 3,923 out-patients. The total income of the year had been £2,898, while the expenditure was £3,743. Patients in the free beds and their friends had during the year contributed £225.

DURING last year the gross amount received by the Royal Victoria Hospital, Belfast, from the Hospital Saturday collection was £609; the expenses of this collection were £58, leaving a net balance of £551. The subscriptions to this hospital from the working class amounted to £2,696, against £2,604 in the previous year.

THE new building of the Rosehill Hospital for Children, Babbacombe, will provide accommodation for 14 cots, with the requisite rooms for matron and nurses, an operating room, a dining room, baths and lavatories. An emergency ward for two beds is also provided. The approximate cost is £2,300, the bulk of which has already been subscribed. Any donor of £25 is entitled to a "name cot" for one year, and to decide about cases which apply for it. Since the hospital was opened in 1888 over 300 cases have been admitted.

### THE STUDENT OF MEDICINE.

### APPOINTMENTS.

**Geo. W. W. Ashdown**, M.D.Edin., appointed District Medical Officer of the Belper Union. **H. H. Ballachey**, L.R.C.P.Edin., M.R.C.S.Eng., appointed District Medical Officer of the Sleaford Union. **O. Bjornson**, M.D., C.M.Manitoba, appointed Clinical Assistant to the Chelsea Hospital for Women. **B. J. Brandson**, M.D., C.M.Manitoba, appointed Clinical Assistant to the Chelsea Hospital for Women. **Maynard Horne**, M.A., M.B., B.C.Cantab., M.R.C.S., L.R.C.P., appointed Assistant Anæsthetist to the National Orthopædic Hospital, Great Portland Street, W. **Miss Jessie S. B. Hunter**, M.B., appointed Assistant Medical Officer of the Lawn Hospital for Insane, Lincoln. **H. Foster Jeffery**, M.B., Ch.B.Vict., appointed Honorary Surgeon to Bury Infirmary and Police Surgeon for Heywood. **J. E. Judson**, M.R.C.S., L.R.C.P.Lond., appointed House Surgeon to the Ashton-under-Lyne Infirmary. **J. M'Gregor**, M.D.Glasg., appointed Medical Officer to the Kyle Union Poorhouse Board. **J. A. Matson**, B.A., M.D.Univ. Dub., L.R.C.P.I., appointed Assistant Physician to the Richmond, Whitworth, and Hardwicke Hospitals. **J. T. Moore**, L.R.C.P., L.R.C.S.Edin., appointed Assistant Medical Officer to the Liverpool Parish Workhouse. **Arthur Latham Ormerod**, M.D., D.P.H.Oxon., appointed Medical Officer of Health for the City of Oxford, and Superintendent of the Corporation Hospitals. **R. B. Robson**, M.B.Dur., appointed Medical Officer of Health to the Alnwick Urban District Council. **G. A. Simmons**, M.D.Lond., appointed District and Workhouse Medical Officer of the St. George's Union. **F. E. Taylor**, M.B., Ch.B.Vict., M.A., M.Sc., M.R.C.S., L.R.C.P., appointed Pathologist to the Chelsea Hospital for Women.

### "The Hospital" Institutional Library.

"Hospitals and Asylums of the World." 4 Vols., with a Portfolio of Plans, complete £12 12s.

"Burdett's Hospitals and Charities, 1902." 5s.

"Cottage Hospitals, General, Fever, and Convalescent." 10s. 6d.

"Hospital Expenditure: The Commissariat." 2s. 6d.

"A Legal Handbook for the Use of Hospital Authorities." 2s. 6d.

"The Cottage Hospital Case Book or Register of Patients." 10s. and 12s. 6d.

"The Furnishing and Appliances of a Cottage Hospital." 6d.

All these are published by the SCIENTIFIC PRESS, Ltd., and may be obtained through any bookseller, or direct from the publishers, 28 and 29 Southampton Street, Strand, London, W.C.

